# Intense ^18^F-Flourodeoxyglucose Uptake in Brachial Plexus of Patients with Brachial Plexopathy

**DOI:** 10.4274/mirt.galenos.2019.40469

**Published:** 2020-04-29

**Authors:** Çiğdem Soydal, Pınar Akkuş, Mine Araz, Güngör Utkan

**Affiliations:** 1 Ankara University Faculty of Medicine, Department of Nuclear Medicine, Ankara, Turkey; 2 Ankara University Faculty of Medicine, Department of Medical Oncology, Ankara, Turkey

**Keywords:** Brachial plexopathy, metastatic breast cancer, 18F-fluorodeoxyglucose positron emission tomography

## Abstract

Brachial plexopathy is a significant cause of pain and disability in patients with breast cancer. Major causes of brachial plexopathy in patients with breast cancer are metastatic invasion or radiation damage to the plexus. Differentiation between the two pathologies is important for appropriate treatment planning. The complicated anatomy of the plexus makes this a difficult area to image accurately. Magnetic resonance imaging (MRI) is the imaging modality of choice for diagnostic evaluation of these cases. We presented a case to demonstrate the role of ^18^F-flourodeoxyglucose positron emission tomography/computerized tomography for confirming metastatic brachial plexopathy when MRI findings were suspicious and for differentiating radiation-induced brachial plexopathy from metastatic plexopathy.

## Abstract (tr)

Brakial pleksopati, meme kanserli hastalarda ağrı ve hareket kısıtlılığının önemli bir nedenidir. Brakial pleksopatinin en sık görülen nedenleri metastatik invazyon ve radyasyon hasarıdır. Tedavi planının doğru yapılabilmesi için bu iki sebebin ayrımı önemlidir. Pleksusun karışık anatomisi nedeniyle bu bölgenin doğru görüntülenmesi zordur. Bu olgularda tanısal değerlendirme için tercih edilen görüntüleme yöntemi manyetik rezonans görüntülemedir (MRG). MRG’nin şüpheli olduğu durumlarda metastatik brakial pleksopati tanısını koymada ve radyasyona bağlı pleksopati ile metastatik pleksopatinin ayrımında ^18^F-florodeoksiglikoz pozitron emisyon tomografi/bilgisayarlı tomografi’nin önemini gösteren bir olgu takdim ediyoruz.

## Figures and Tables

**Figure 1:**
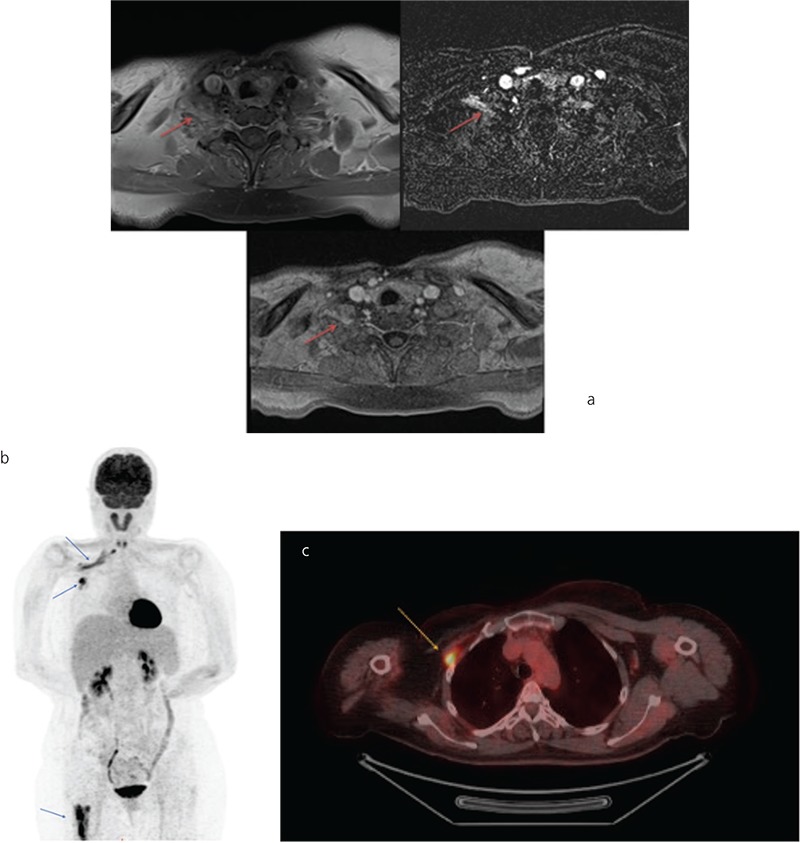
A 50-year-old woman diagnosed as having infiltrating ductal carcinoma of the right breast had undergone surgery (modified radical mastectomy and lymph node dissection), followed by adjuvant chemotherapy and radiotherapy six years ago. She presented to her oncologist with pain and restriction of movement of the right upper limb lately. Magnetic resonance imaging (MRI) was used for evaluation of brachial plexus and minimal thickening of plexus nerves was detected, which could be caused by radiation damage (Figure 1a, red arrows). The patient was referred for ^18^F-fluorodeoxyglucose (FDG) positron emission tomography/computerized tomography (PET/CT) for restaging of disease after it was observed that tumor marker level increased. ^18^F-FDG PET/CT was performed and maximum intensity whole body and fused transaxial images of thorax revealed linear pattern of pathological FDG uptake in the right lateral aspect of the upper chest, right axillary and interpectoral lymph nodes and right femur (Figure 1b and c, blue and yellow arrows).

**Figure 2:**
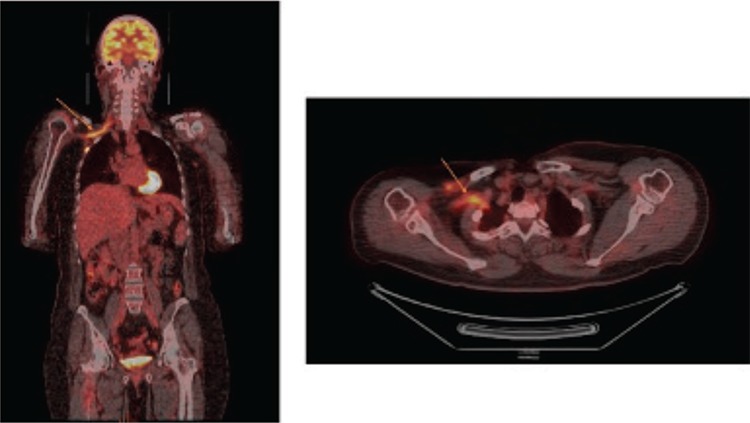
The coronal and transaxial fused PET/CT images revealed a linear pattern of FDG uptake in right brachial plexus (orange arrows). Brachial plexopathy is a rare condition with an incidence of less than 0.5%. Metastatic breast and lung cancers are the most common nontraumatic causes of brachial plexopathy following radiation induced plexopathy (1). MRI is the modality of choice for diagnosis of brachial plexopathy and preferred to other imaging modalities for characterization of brachial plexopathy due to its multipl planar capabilities and superior soft-tissue contrast. In MRI, radiation fibrosis shows diffuse thickening and minimal enhancement along the brachial plexus and MRI cannot always readily differentiate radiation-induced plexopathy from metastatic plexopathy (2). ^18^F-FDG PET/CT is a useful tool in the evaluation of patients with suspected metastatic plexopathy, particularly when MRI findings are suspicious (3). The typical pattern as seen in MIP and coronal images is linear uptake, extending from the superomedial aspect (supra/infra clavicular) to the lateral aspect of axilla closely related to the subclavian/axillary vessels (4). ^18^F-FDG PET/CT plays an important role in diagnosing neoplastic plexopathy, differentiating it from radiation-induced plexopathy and monitoring response to treatment.
